# Supplementary material for: Mexican and Spanish university students’ Internet addiction and academic procrastination: Correlation and potential factors
Source: PLoS One. 2020 May 22;15(5):e0233655. doi: 10.1371/journal.pone.0233655 (PMC7244110; doi:10.1371/journal.pone.0233655)
Supplement: Attachment — Submitted filename: Comments_PLOS ONE.pdf [file pone.0233655.s002.pdf]

I much appreciate the opportunity to review the manuscript entitled “Internet addiction and academic procrastination in Mexican and Spanish university students. Correlation and Predictive Factors” This study examined the prevalence and sociodemographic correlates of internet addiction, and further ascertained the association between internet addiction and academic procrastination in college students from both Spain and Mexico. I read this manuscript with great interest, and I believe this study involves some possible strengths, such as a cross-cultural design, and focusing on a growing public health concerns in society. In this regard, I believe addressing these research questions may have some potential contributions to society (not only in the two countries under study but also in a worldwide perspective). Along with these positive evaluations, I have some pressing concerns and possible suggestions, as I listed below. Hopefully, these comments are found to be helpful to potentially improve the quality of the manuscript.

1. Considering the cross-sectional nature of this study, please omit “predict” and “predictive” throughout the manuscript. This is because such a design cannot infer the causal linkage under investigation. Please also elaborate more on this issue in the Limitations (although the authors only refer to the correlation in SEM model).

2. The manuscript should be edited by a professional native speaker, as many grammatical errors and uncommon phrases have been identified throughout the paper.

3. What does it mean by university students exactly? Were all undergraduate, postgraduate, or doctoral students considered? Why is the sampling so broad? Concerning the means and standardized deviations of the table, it indicates that participants across two countries aged 47 and 58 years are considered as possible outliers.

4. 13. In the introduction, the authors always shift among high school students, adolescents, and university students? Since high school students and adolescents are not the focus of this study, please be selective and concentrate when conducting the literature review.

5. 12. Introduction, the authors mentioned, “this problem has spread globally in developed countries” This is inaccurate, as the similar patterns have also been documented in many developing countries.

6. Are these two selected universities comparative in terms of socioeconomic status and study background? It may be the case that, before this investigation, they have already shown some inherent differences.

7. How do the authors ensure the cross-cultural equivalence of the measurement without running multi-group CFA? Relatedly, I was wondering why the authors do not conduct MG-SEM to investigate any association differences between the two countries.

8. Based on the table 1, gender is not fully balanced, particularly in the subsamples of participants from Spain. Why? Moreover, several sociodemographic characteristic are not fully balanced; instead, the authors do not consider including these as confounding variables.

9. Please add the item examples for each questionnaire (PP. 10-11).

10. Please elaborate more on the rationale of selecting these model fit indices (P. 12).

11. What does it mean by gl exactly? (P. 13)

12. What is RMR? May you indicate SRMR? (P. 15)

13. Is any missing data involved in the present research? How do the authors handle them in the further course of data analysis?

14. When comparing the internet addiction between two countries, the authors fail to consider the sociodemographic variables that may potentially influence the mean level differences. In a sense, MANCOVA should be administrated (P. 13).

15. The fit indices of SEM do not show that the model fits the data well ($X^2/df = 5.63$, and p-value is significant; P. 16). This is a significant concern.

16. Overall, the discussion is poorly addressed by only two pages. I highly encourage the authors to discuss thoughtfully and more in depth concerning each purpose of this study.

17. Please add the new section of Limitations and Implications, and remove the limitations from the conclusion section. Limitations are not conclusions; rather, they should be addressed in the discussion section.

18. The figure provided is unclear. Moreover, according to this figure, some factor loadings are inappropriate. Why are they still being considered in further analysis?
